# Evaluation of research on interventions aligned to WHO ‘Best Buys’ for NCDs in low-income and lower-middle-income countries: a systematic review from 1990 to 2015

**DOI:** 10.1136/bmjgh-2017-000535

**Published:** 2018-02-19

**Authors:** Luke N Allen, Jessica Pullar, Kremlin Khamarj Wickramasinghe, Julianne Williams, Nia Roberts, Bente Mikkelsen, Cherian Varghese, Nick Townsend

**Affiliations:** 1 Centre on Population Approaches for NCD Prevention, Nuffield Department of Population Health, University of Oxford, Oxford, UK; 2 Health Library, Nuffield Department of Population Health, University of Oxford, Oxford, UK; 3 Global Coordination Mechanism for Noncommunicable Diseases, WHO, Geneva, Switzerland; 4 Department for Management of Noncommunicable Diseases, Disability, Violence and Injury Prevention, WHO, Geneva, Switzerland

**Keywords:** non-communicable diseases, best buys, low and lower middle income countries, developing countries

## Abstract

**Background:**

Non-communicable diseases (NCDs) are the leading cause of death and disability worldwide, with low-income and middle-income countries experiencing a disproportionately high burden. Since 2010 WHO has promoted 24 highly cost-effective interventions for NCDs, dubbed ‘best buys’. It is unclear whether these interventions have been evaluated in low-income and lower-middle-income countries (LLMICs).

**Aim:**

To systematically review research on interventions aligned to WHO ‘best buys’ for NCDs in LLMICs.

**Methods:**

We searched 13 major databases and included papers conducted in the 83 World Bank-defined LLMICs, published between 1 January 1990 and 5 February 2015. Two reviewers independently screened papers and assessed risk of bias. We adopted a narrative approach to data synthesis. The primary outcomes were NCD-related mortality and morbidity, and risk factor prevalence.

**Results:**

We identified 2672 records, of which 36 were included (608 940 participants). No studies on ‘best buys’ were found in 89% of LLMICs. Nineteen of the 36 studies reported on the effectiveness of tobacco-related ‘best buys’, presenting good evidence for group interventions in reducing tobacco use but weaker evidence for interventions targeting individuals. There were fewer studies on smoking bans, warning labels and mass media campaigns, and no studies on taxes or marketing restrictions. There was supportive evidence that cervical screening and hepatitis B immunisation prevent cancer in LLMICs. A single randomised controlled trial supported polypharmacy for cardiovascular disease. Fourteen of the ‘best buy’ interventions did not have any good evidence for effectiveness in LLMICs.

**Conclusions:**

We found studies on only 11 of the 24 interventions aligned with the WHO ‘best buys’ from LLMIC settings. Most LLMICs have not conducted research on these interventions in their populations. LLMICs should take action to implement and evaluate ‘best buys’ in their national context, based on national priorities, and starting with interventions with the strongest evidence base.

**Key questions**

**What is already known about this topic?**

- The WHO published a number of highly cost-effective non-communicable disease (NCD) policy options—dubbed ‘best buys’—that are included in the Global Action Plan for the prevention and control of NCDs.
- Low-income and middle-income countries face the greatest burden of NCDs and have been encouraged to implement these polices as a priority.
- The ‘best buys’ are identified from the global evidence base, but it is unclear how many research activities are carried out in low-income and lower-middle-income countries (LLMICs) related to these priority NCD actions.

**What are the new findings?**

- There is a general lack of published evidence for the ‘best buy’ interventions in LLMICs, and a number of the interventions have not been evaluated at all in these settings.
- Most of the existing research is concentrated in South-East Asia. Our search only returned five studies from the African region, two from the Eastern Mediterranean, two from South-East Asia, one from the Americas, and none from the European or Western Pacific regions.
- More than half of the identified studies evaluated tobacco-related interventions.

**Recommendations for policy**

- This study highlights the need for prioritising NCD ‘best buys’ in national research agendas in LLMICs. This would contribute to the generation of more ‘context specific’ evidence for NCD prevention and control, and improve the implementation of those policies.
- Countries that introduce ‘best buys’ should try to evaluate these interventions and publish findings in the public domain.

## Introduction

Non-communicable diseases (NCDs) include cardiovascular disease, chronic respiratory diseases, cancers and diabetes. They are the leading cause of morbidity and mortality, accounting for 68% of global deaths in 2012, of which 16 million occurred in people younger than 70 years.^1^ The financial repercussions of NCDs are commensurate with the global burden, representing an estimated $47 trillion in lost output to the global economy by 2030.^2^ Previously conceived of as ‘diseases of affluence’, the risk of premature death is actually highest in low-income and middle-income countries.^3^

The inclusion of NCDs in the 2015 ‘Sustainable Development Goals’ acknowledges both the outsized global impact of NCDs and the unequal distribution of human and economic costs. Governments have committed to reducing premature NCD mortality by a third^4^; however, many policymakers have been unsure where to prioritise their efforts. Responding to these concerns, WHO identified a number of highly cost-effective NCD policy options, dubbed ‘best buys’ (table 1). These interventions, including tobacco taxation, salt reduction and cervical cancer screening, were first outlined in the 2010 WHO Global Status Report on NCDs and have been endorsed by the World Economic Forum and Harvard School of Public Health.^5–7^

**Table 1:** Interventions used in this review

Risk factor/disease	WHO ‘best buy’	Specific interventions
Tobacco	Raise taxes on tobacco	Introduce or increase excise taxes
	Protect people from tobacco smoke	Ban smoking in public places
	Enforce bans on tobacco advertising	Advertising/promotion/sponsorship bans
	Warn about the dangers of tobacco	Information and warnings on tobacco packaging
		Mass media campaigns
		Group smoking reduction programmes
		Individual programmes
Unhealthy diet and physical inactivity	Mass media campaigns—physical activity	Evidence-informed campaigns on activity
	Mass media campaigns—diet	Evidence-informed campaigns on diet
	Replace trans fat with polyunsaturated fat	Reformulation
		Labelling
		Mass media campaigns
	Reduce salt intake	Mass media campaigns
		Reformulation
Harmful alcohol use	Raise taxes on alcohol	Introduce or increase excise taxes
	Restrict access to retailed alcohol	Regulating commercial and public availability*
	Enforce bans on alcohol advertising	Advertising/promotion bans
Cardiovascular disease	Counselling and polydrug therapy for high-risk groups†	Prevention: polydrug (≥2 antihypertensives) if BP >160/100
		Prevention: polydrug (≥2 agents) if 10-year CVD risk ≥30%
		IHD/stroke treatment: combination of aspirin+B blocker+ACE inhibitor
		Diabetes (HbA1c >9%): ≥1 antidiabetic; polydrug Rx if BP >165/95
Cancers	Treat heart attacks with aspirin	Acetylsalicylic acid for acute myocardial infarction
	Hepatitis B immunisation to prevent liver cancer	Hepatitis B immunisation
	Screening and treatment to prevent cervical cancer	VIA/Pap smear with timely treatment of precancerous lesions

*We have included legislative age restrictions on alcohol use as a means of restricting access to retailed alcohol.

†Studies on medical treatment were included even if they did not include a counselling component.

BP, blood pressure; CVD, cardiovascular disease; HbA1c, haemoglobin A1c; IHD, ischaemic heart disease; Rx, therapy; VIA, visual inspection with acetic acid.

Although the reasoning behind the ‘best buys’ is relatively uncontroversial, it is possible that context-specific factors may influence the effectiveness of the ‘best buys’ in low-income and lower-middle-income countries (LLMICs). This is important because LLMICs bear the greatest burden of premature NCD deaths.^8^ The evidence for many of the interventions comes from high-income countries and may not be generalisable to low-income settings. As the WHO ‘best buys’ represent the de facto global strategy to control NCDs, it is important that these interventions work, especially in populations facing the highest burden disease.

We aimed to systematically review studies evaluating ‘best buy’ interventions in LLMICs in order to establish whether all of the ‘best buys’ have been evaluated in these settings, how much research has been conducted on each intervention and where the research has been performed.

## Methods

### Identification and selection of studies

Using a registered protocol (PROSPERO: 42016039051) and following the Preferred Reporting Items for Systematic Reviews and Meta-Analyses (PRISMA) guidelines^9^ (online supplementary material), we identified records published from 1 January 1990 to 5 February 2015, searching MEDLINE, Embase, Global Health, Ovid MEDLINE and Web of Science Core Collection. We also searched grey literature in Digital Dissertations, the WHO Global Health Library Regional Index and the first 100 hits from Google Scholar. We scrutinised reference lists and contacted key authors to uncover additional or forthcoming work.

10.1136/bmjgh-2017-000535.supp1 Supplementary file 1

The same dates and strategy (online supplementary appendix 1) were used for all searches, tailored to specific databases by an experienced medical librarian. We conducted the search in English but did not restrict results by language or age of participants.

10.1136/bmjgh-2017-000535.supp2 Supplementary file 2

### Study selection

Studies were included if they provided quantitative evaluation of one or more ‘best buy’ interventions based in one or more of the 83 LLMICs, as defined by the 2014 World Bank analytical classifications (fiscal year 2016).^10^ Studies were included if they reported primary quantitative data on outcomes relating to NCD behavioural risk factors, morbidity or mortality, for instance smoking quit rates or systolic blood pressure. As the first scoping review on this topic, we wanted to include all research on ‘best buys’, so we also included studies reporting process indicators and proximal measures such as changes in awareness and knowledge, and self-reported intentions, for example intention to quit smoking.

We excluded reviews, editorials and studies that could not be used to quantify the impact of ‘best buy’ interventions, that is, those that did not provide two or more sets of outcome data to allow comparison before and after the instigation of a ‘best buy’, or between exposed and unexposed groups. In practice this excluded case series and traditional cross-sectional studies that only provide outcome data for one population at one point in time. We excluded studies that compared two different forms of the same intervention without reference to a control/usual care group, for example studies comparing the effect of two different polypills on hypertension. We had the resources to translate studies written in English, French, Spanish, Portuguese, Italian and Sinhalese. Papers that were written in any other language were excluded.

We defined the ‘best buy’ interventions as the 24 interventions that were presented in the 2011–2020 NCD Global Action Plan^11^ and the supporting WHO discussion paper (summarised in table 1).^6^

Using a piloted form JP and LA independently screened titles and abstracts, calculating percentage agreement and Cohen’s kappa statistic at 10% intervals (every 267 records). Once inter-rater agreement exceeded 95% and Cohen’s kappa >0.75 (‘excellent’ agreement^12^), JP screened all remaining records. Uncertainties were resolved by group consensus. The same protocol was used for full-text review. Authors were contacted by email if more information was required.

### Data extraction and quality assessment

JP and LA used a piloted version of the Cochrane Effective Practice and Organisation of Care (EPOC) data collection checklist^13^ to independently extract relevant data, including study type, population, intervention, comparator, methods, outcomes and results (see online supplementary appendix 2 for a full list of extracted variables). KKW and NT independently cross-checked a random 10% sample of included papers. Disagreements were resolved by group consensus.

We assessed the risk of bias for randomised controlled trials (RCTs) using the Cochrane Collaboration’s tool.^14 15^ We used the EPOC criteria to assess risk of bias in interrupted-time-series studies, and we used study-specific versions of the Newcastle-Ottawa Scale^16^ for all other non-randomised trials, as recommended by the Cochrane Collaboration.^14^ Scores from the latter two instruments were based on selection methods, comparability and outcome reporting, and were reported as ‘low’, ‘medium’ or ‘high’ risk of bias (online supplementary appendix 3).

We graded level of evidence (1=high, 5=low) using a modified scheme from the Oxford Centre for Evidence-based Medicine (online supplementary appendix 4).

### Synthesis

Variation in the interventions and outcome measures of different studies precluded quantitative synthesis and meta-analysis. We adopted a narrative approach, grouping studies by intervention category.

## Results

**Figure 1:**
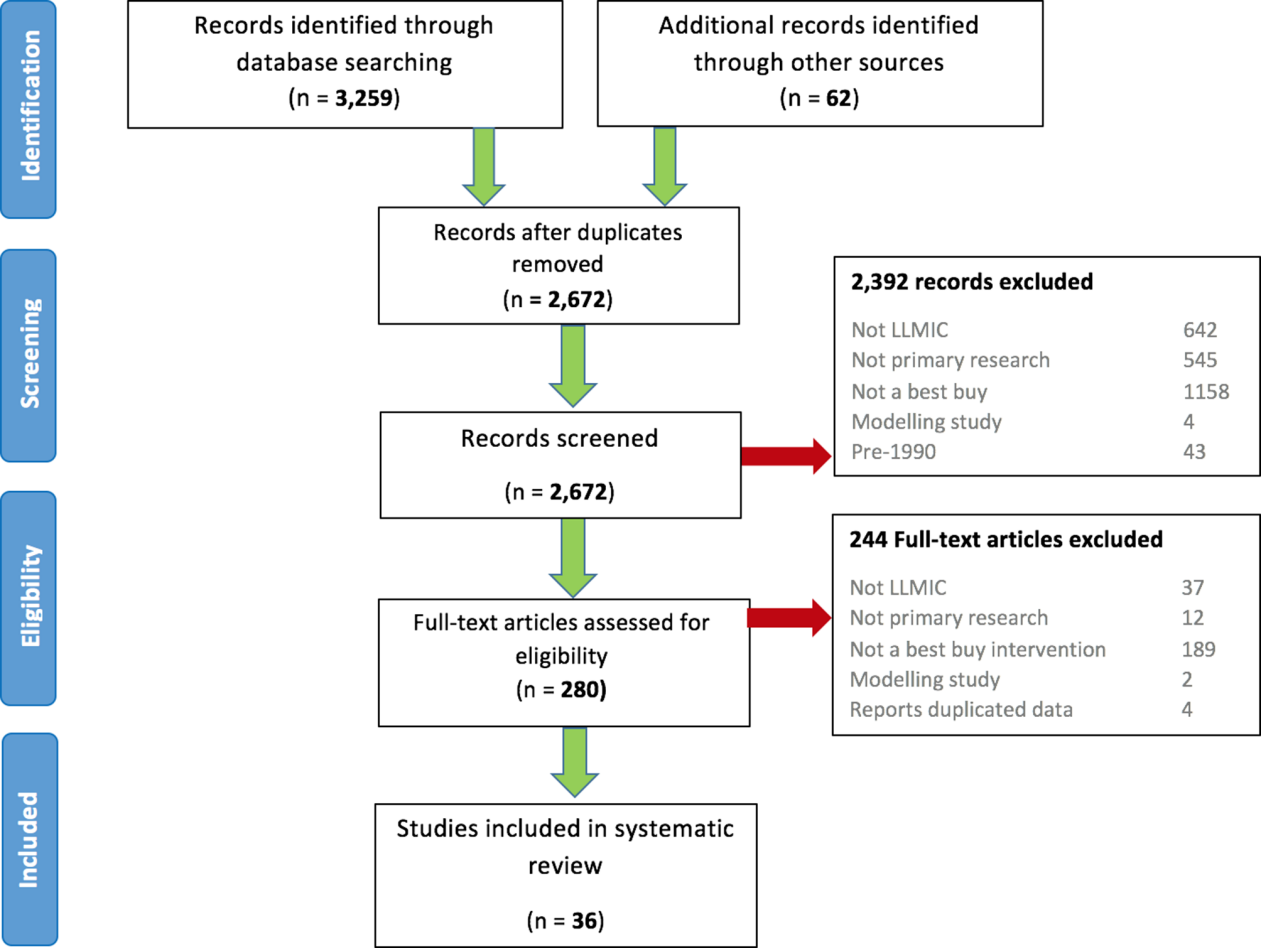
Preferred Reporting Items for Systematic Reviews and Meta-Analyses diagram. LLMIC, low-income and lower-middle-income country.

Our initial search returned 2672 records. After excluding 2392 records based on title and abstract screening, 280 papers were included for full-text review (figure 1).

**Figure 2:**
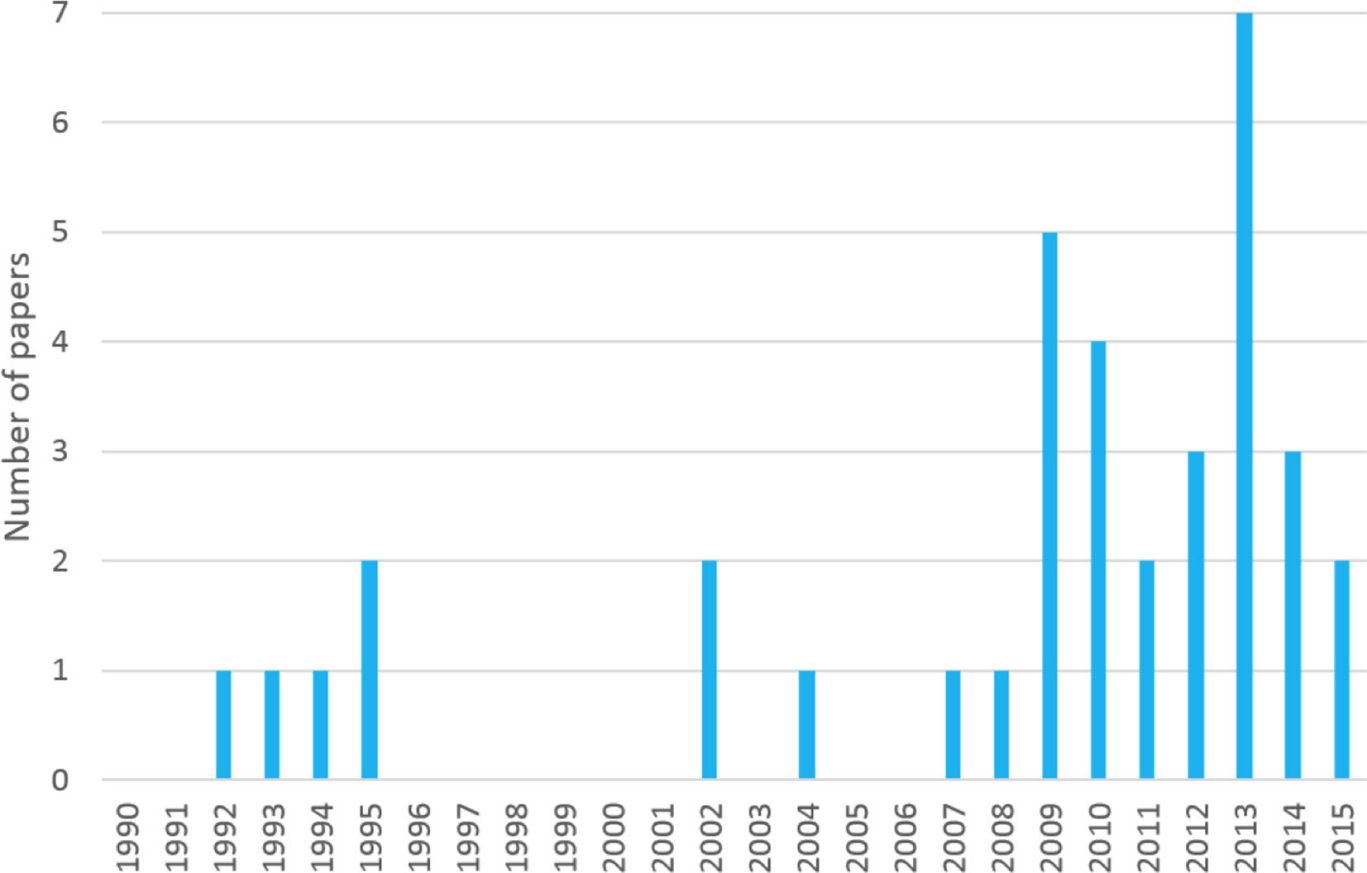
Number of studies published each year, 1990–2015.

Thirty-six studies (608 940 participants) were included: 19 RCTs (of which 3 were phase II drug trials), 7 cohort studies, 6 longitudinal studies and 4 retrospective cross-sectional studies. Eight of the studies were published before 2007 (figure 2).

There were no studies from 74 of the 83 LLMICs (89%). Our search returned five studies from the WHO African region, two from the Eastern Mediterranean, 28 from South-East Asia, one from the Americas, and none from the European or Western Pacific regions.

Almost three-quarters of the studies came from India. Of the 26 Indian studies, 17 examined tobacco-related ‘best buys’, 5 looked at cancer and 4 examined ‘best buys’ related to cardiovascular disease. Single studies from Indonesia and Bangladesh evaluated cervical cancer screening and a group smoking cessation programme, respectively.

**Figure 3:**
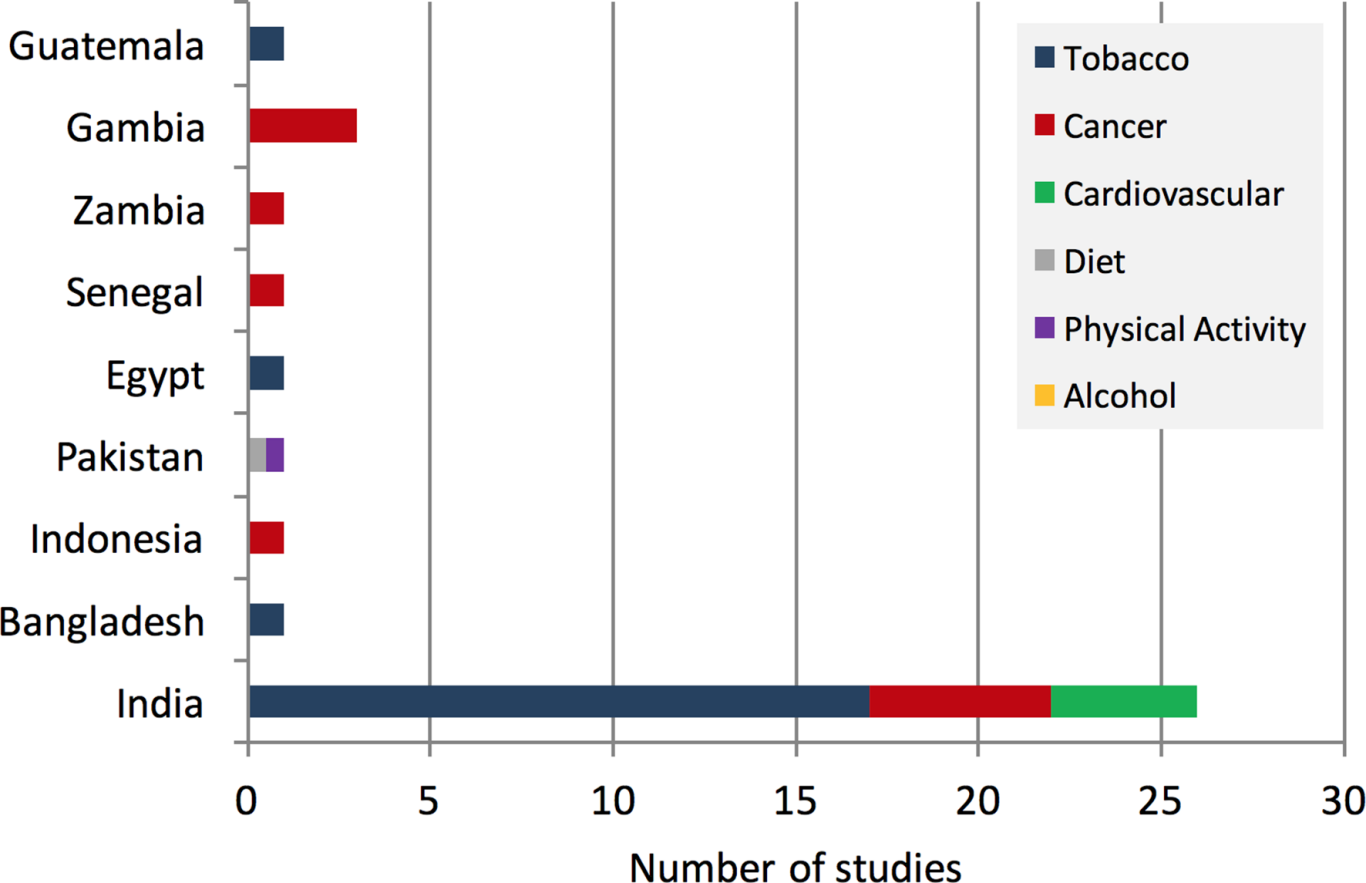
Country of origin of included studies.

A single Pakistani study examined the effectiveness of a mass media campaign on diet and physical activity. The single Egyptian study examined group smoking cessation; the Guatemalan paper examined longitudinal air quality measurements before and after a national smoking ban; and the studies from Senegal, Zambia and Gambia all examined cancer interventions (figure 3).

In total, 19 of the 36 studies reported on the effectiveness of tobacco-related ‘best buys’ in LLMICs^17–35^; 2 on physical activity and diet^36 37^; 4 on cardiovascular disease^38–41^; and 11 on cancer.^42–52^

**Figure 4:**
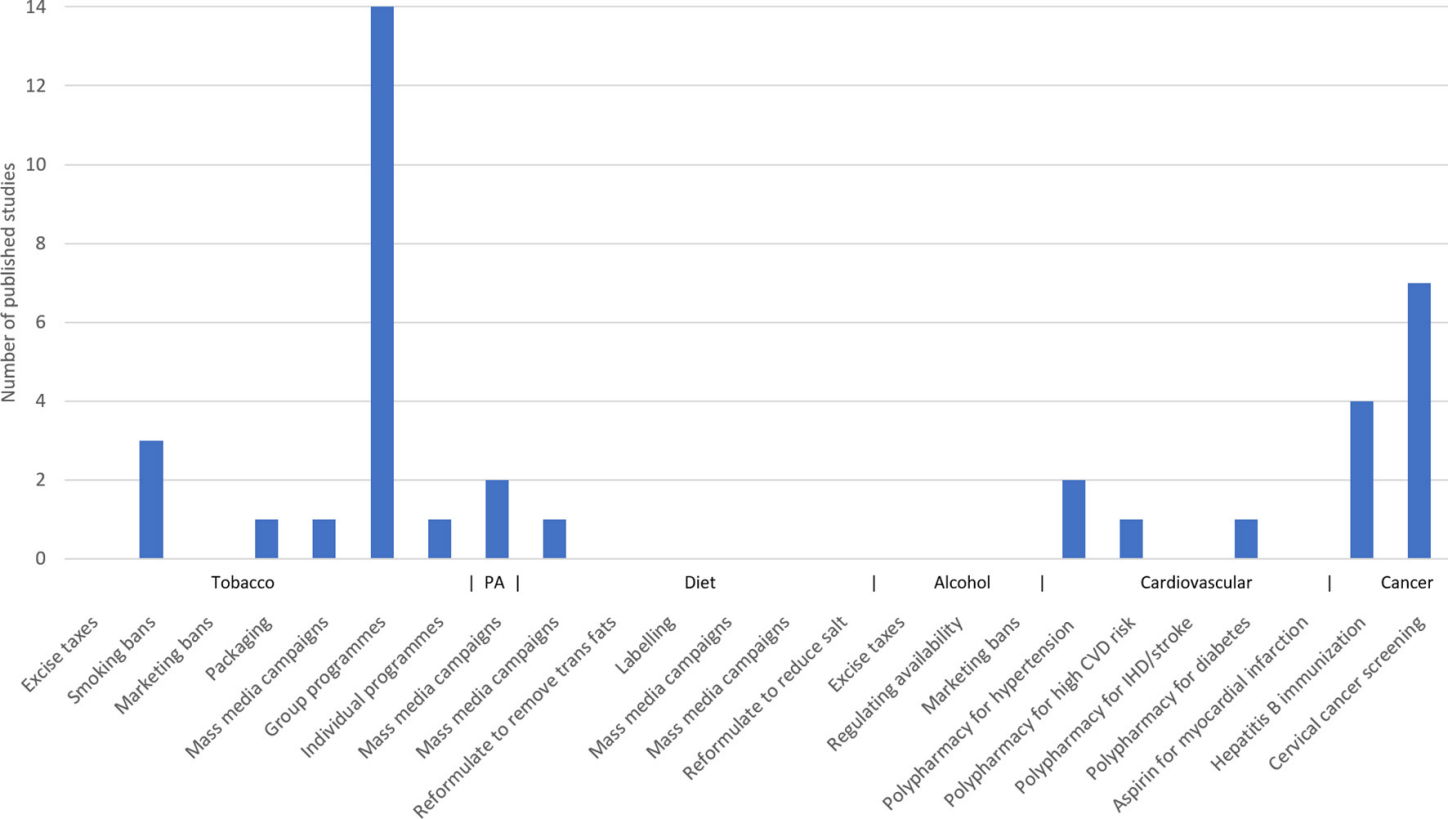
Number of studies for each intervention. CVD, cardiovascular disease; IHD, ischaemic heart disease; PA, physical activity.

Many of the ‘best buy’ interventions within each category had not been evaluated at all; there were no studies evaluating tobacco taxation or marketing restrictions, and no studies evaluated polypharmacy for ischaemic heart disease or aspirin for myocardial infarction. There were no studies on any of the trans fat, salt or alcohol ‘best buys’ (figure 4).

Of the ‘best buys’ that were examined, each had at least one study that provided evidence for effectiveness. Savant *et al* did not present a measure of statistical significance for their findings, but all other papers presented effect sizes that were all significant at the 0.05 level. An overview of study characteristics and findings is presented in online supplementary table 1.

10.1136/bmjgh-2017-000535.supp3 Supplementary file 3

Many of the studies used process indicators or proximal incomes rather than ‘harder’ endpoints; for instance, a survey of Indian bus drivers found that those who had been exposed to graphical tobacco warnings had better knowledge of the risks of tobacco than those who had not seen the warnings.

Despite the tendency towards measuring ‘soft’ outcomes, the studies were generally well-conducted: 20 were rated as low risk of bias, and the remaining 16 were rated medium risk using our scoring rubric (online supplementary appendix 3).

The RCTs tended to be well-conducted. Each one used random sequence allocation and almost all obtained complete outcome data; however, selective reporting and lack of blinding were common limitations. RCTs were exclusively used to evaluate tobacco cessation programmes, polypharmacy for cardiovascular disease, and cervical cancer screening and treatment. As such these interventions have the highest grade of evidence.

All three studies evaluating bans on tobacco use in public places adopted longitudinal approaches, as did two group smoking cessation evaluations and a cervical cancer screening evaluation. Only one of these studies adequately addressed incomplete data. Two of the longitudinal studies failed to report all of the outcomes mentioned in the methods. Longitudinal studies are not good at controlling for confounding and therefore represent relatively low-grade evidence.

The cohort studies all used representative samples, and all but one ascertained exposure adequately. The most common source of bias was inadequate follow-up. All but one of the cohort studies examined either cervical screening or hepatitis B interventions. Prospective cohort studies offer a higher grade of evidence than longitudinal studies; however, they are inferior to RCTs as they are still vulnerable to selection bias.

Finally, the retrospective cross-sectional surveys were used to ask respondents if they felt that the introduction of smoking bans, tobacco warning labels, and mass media campaigns on tobacco, diet and physical activity had been effective. All of these studies tended to be very well-conducted with adequate and representative sample sizes and appropriate outcome assessments and statistical tests. Nevertheless, retrospective cross-sectional surveys represent a lower grade of evidence than RCTs and prospective cohort studies.

## Discussion

This systematic review is the first attempt to assess whether the WHO’s prescription for the NCD pandemic has been evaluated in settings that bear the greatest burdens of death and disability. Only half of the 24 interventions that have been designated ‘best buys’ have been evaluated in LLMICs.

Three-quarters of the evaluations have been conducted in South-East Asia, and our search did not return any studies from Western Pacific or European LLMICs.

Only six of the interventions have had two or more studies that evaluate effectiveness: smoking bans in public places, group smoking reduction programmes, physical activity mass media campaigns, counselling and polypharmacy for high cardiovascular risk groups, cervical cancer screening, and hepatitis B immunisation.

Five of the ‘best buys’ have only been evaluated by single studies: tobacco labelling, tobacco mass media, diet mass media, polypharmacy for high cardiovascular risk groups and polypharmacy for diabetics. Our review was not designed to assess the effect sizes of each ‘best buy’, and there was significant heterogeneity in study designs and outcome measures.

Group smoking reduction programmes had the highest quantity and quality of evidence, with 12 RCTs (grade 1 evidence) assessed as medium and low risk of bias. Pharmacology trials were of a similar quality. Studies examining the other ‘best buys’ tended to be well-conducted but of a lower grade (prospective cohorts, retrospective cross-sectional surveys and longitudinal studies).

The studies were generally well-conducted, with around two-thirds of studies being rated as high quality within each study design grouping. The grade of evidence was highest for individual-level tobacco interventions.

The population-based nature of many of the ‘best buys’ makes it difficult to randomise and blind participants. A number of trials successfully used interrupted time-series approaches, cluster randomisation and cohort designs to overcome this issue. Cluster RCTs represent the highest level of evidence but are vulnerable to bias from imbalance between the study arms. They are also less precise than individual-level RCTs.

Considering the individual ‘best buy’ interventions in more depth, there was no evidence for any of the alcohol ‘best buys’ in LLMICs. Alcohol abstention rates are high in low-income countries, and in some settings alcohol use is so low that the interventions may not be necessary. As a broader point, interventions need to be tailored to the local context and all of the ‘best buys’ do not necessarily need to be applied in every setting. Having said this, a number of LLMICs do have high rates of alcohol use, and the relative alcohol-related disease burden tends to be highest among low-income populations.^53^ Cook *et al*^54^ provide evidence that restricting availability to alcohol through licensing, age restrictions, higher pricing and advertising restrictions all reduced consumption levels in an analysis of 15 low-income and middle-income countries. Furthermore LLMICs are increasingly being targeted by alcohol companies, and aggressive marketing strategies have been reported in Gambia, Ghana, Nigeria and Uganda.^55^ Effective alcohol harm reduction polices are urgently needed in these settings, including policies that target informally retailed products.^56^

Tobacco use is steady or declining in all WHO regions except for the African region, and more cigarettes are smoked in China than in all LLMICs.^57^ However the number of tobacco outlets is 2.5 times higher in low-income countries than in high-income or middle-income countries.^58^ Although the number of countries employing ‘best buy’ tobacco policies is increasing, the absolute number remains low,^59^ and our findings suggest that a minority of policies are being scientifically evaluated.

While tobacco excise taxes have been implemented in 12 LLMICs, there is no published evidence to show that they are effective in these countries.^60^ Two modelling studies have suggested that excise taxes could significantly reduce smoking prevalence in Vietnam.^61 62^

Three studies demonstrated that smoking bans in public places reduced air nicotine levels. While encouraging, future research should examine the impact of smoking bans on clinical outcomes. To date 15 LLMICs have implemented smoking bans policy, led by Nepal, Burkina Faso, Chad and Madagascar.^60^ The Framework Convention on Tobacco Control is clear that all indoor public places, public transport facilities and indoor workplaces should be 100% smoke-free,^63^ but only 18% of the world’s population is currently covered by this level of legislation.^59^ While modelling studies suggest that public smoking bans are highly effective,^64^ enforcement issues highlight the need for comprehensive policy measures and implementation.

No studies reported on tobacco marketing restrictions, but a global study found that consumption levels correlate inversely with national bans.^65^ Surveys by Savell *et al*^58^ show that exposure to tobacco marketing is 10 times higher in low-income compared with high-income countries.

Many LLMICs have successfully introduced health information and warnings on tobacco packaging, but there is only one study assessing effectiveness. In recent years Samoa, the Philippines, Bangladesh, the Solomon Islands, Vanuatu and Vietnam have implemented large graphic pack warnings, but there have not been published evaluations to date. Pant *et al* have shown that it is difficult to enforce pictorial health warnings where tobacco products are produced in the informal sector, as is the case for most LLMICs. This limits the generalisability of evaluations from settings like Australia and the UK, where the informal sector has a much smaller market share.^66^

Only one study examined the impact of a tobacco mass media campaign in an LLMIC; however, this policy is thought to be one of the most cost-effective.^67^

Fourteen studies reported the effectiveness of group tobacco reduction programmes, predominantly based in communities and schools and using a mixture of education, counselling and group activities. Most were limited by reliance on self-report; however, one study found 96% agreement between self-report and urine cotinine levels. These largely positive findings contrast with an analysis of 43 countries (including non-LLMICs) that found no independent association between smoking behaviour and exposure to school-based group counselling.^68^

There was no evidence for five of the six dietary interventions. Moldova, the Kyrgyz Republic and Uzbekistan have implemented policies to eliminate trans fats, as has India; however, a 2015 evaluation found high levels remain in street food and household snacks.^69^ More evidence is needed here.

The included Pakistani newspaper-based mass media campaign provided supportive evidence for national campaigns that corroborates modelling studies from Vietnam, Syria and the occupied Palestinian territories.^70 71^ Further modelling studies have shown that modest salt intake reductions are associated with large health gains.^72^ By 2015 five LLMICs had fully implemented salt reduction policies, but none of these were low-income countries.^60^ As processed foods are less important sources of salt in LMICs compared with richer countries, interventions aimed at industrial reformulation are likely to be less effective in these settings.^73 74^

The 2015 WHO NCD progress monitor shows that 33 LLMICs have implemented at least one recent national public awareness programme on diet and/or physical activity.^60^ In high-income countries the evidence that mass media can reduce NCD risk factors is limited. A 2013 systematic review found that campaigns can increase moderate walking but do not significantly increase the amount of people meeting activity recommendations.^75^ Another systematic review including non-LLMICs showed that many diet-related campaigns currently focus on undernutrition rather than overnutrition.^76^

Four studies examined the effectiveness of cardiovascular ‘best buys’. The WHO’s tight definitions for these interventions are likely to have excluded a number of papers; however, it is still surprising that our search returned such a low number of studies.

Only the LLMICs Ukraine, Armenia and Vanuatu routinely provide preventive polypharmacy for cardiovascular disease.^60^ Modelling studies on cardiovascular medicines predict large gains to population health in LLMICs: Lim *et al*^77^ project that cardiovascular polypharmacy could avert 17.9 million deaths in LMICs at a cost of US$0.75–1.30 per capita, but there is little convincing experimental evidence at this stage.

Both hepatitis B immunisation and cervical cancer screening had multiple evaluations with well-conducted and high-grade studies. Ginsberg *et al*^78^ estimate that a single smear test at age 40 years with lesion removal and cancer treatment would avert 462 Disability Adjusted Life Years (DALYs) per million people in the sub-Saharan Africa region and 1327 DALYs per million people in the South-East Asia region at a cost of Int$307 and Int$142 per DALY, respectively.

### Study strengths and limitations

To the best of our knowledge, this is the first review to systematically assess the evidence on whether the WHO ‘best buy’ interventions have been evaluated in LLMICs. Strengths of this study include our adherence to PRISMA and Cochrane guidance and our use of multiple researchers to ensure high levels of agreement at each stage of the review. Following the exact wording of the ‘best buys’ may have restricted our results, especially for cardiovascular therapy; however, our attention to the exact WHO wording is also a strength of this study. The comprehensiveness of our search strategy is another strength.

We set out to ascertain which interventions were being evaluated and where the research was occurring rather than examining effect sizes. Future research is needed to establish how effective the interventions are in LLMICs. Our efforts to find and include all studies on the ‘best buys’ also introduced marked heterogeneity in study designs and outcome measures. This is appropriate for an initial scoping review but limits the ability of the findings in terms of making intervention-specific recommendations. The mix of study types necessitated the development of a new quality scoring rubric that would allow cross-comparability. We used the current gold-standard Newcastle-Ottawa and Cochrane Collaboration tools; however, our composite risk of bias scheme has not been previously validated. To reduce chances of introducing bias, we present raw scores alongside quality ranking in online supplementary table 1. Our search was conducted in 2015 at the eve of the Millennium Development Goals. A major strength of the paper is that 25 years’ worth of global research is presented, ending at a natural transition point in global health policy. The lack of evidence from 2015 to present stems from delays in preparing the manuscript and lack of resources. The omission of recent research weakens our findings.

## Conclusion

The major finding of this systematic review is the widespread paucity of research on ‘best buys’ in LLMICs. The rising burden of NCDs in less developed countries has been overlooked for decades^79^; however, it is surprising that so many well-established interventions lack published evaluations in the areas where the burden of disease is highest. Although aspirin is likely to work in Djibouti just as well as it works in Denmark, the same is not necessarily true for media campaigns, marketing restrictions and taxation policies given heterogeneity in cultural norms and market factors. As premature mortality is highest in low-income settings, it is important that the major NCD interventions are evaluated in these settings. There is an urgent need for implementation research on the diet and alcohol-related ‘best buys’ in LLMICs as the evidence base is so scant.

A number of LLMICs had implemented ‘best buys’ between 1990 and 2015 but did not evaluate effectiveness. Moving forward, all countries should consider evaluating these interventions and publishing findings in the peer-reviewed literature as the corpus of evidence represents a global public good that can be used by governments in deciding how to allocate resources most effectively in order to combat NCDs.

This review showed that hepatitis B immunisation, cervical cancer screening, smoking bans and group smoking reduction programmes are supported by a number of high-quality studies. In the absence of evidence for other interventions, there is an argument that these ‘best buys’ should be prioritised, as long as there is clinical need.

Future research should try to quantify the effect sizes of the various interventions in different settings, employing high-grade study designs. Step-wedge and cluster RCTs can be used to assess population-level interventions, as well as carefully designed longitudinal analyses.

The list of ‘best buys’ could be used to help NCD researchers prioritise their research agenda. WHO has developed an implementation research guide that facilitates evaluation of these interventions in LLMICs.^80^ South-East Asia has been very active in this space, but the other WHO regional offices could do more to promote evaluation and implementation research on these interventions.

## References

[1] World Health Organization. Global Status report on Noncommunicable Diseases, 2014 http://www.who.int/nmh/publications/ncd-status-report-2014/en/ (cited 07 Mar 2016).

[2] Bloom D, Chisholm D, Llopis E, et al From burden to" best buys": reducing the economic impact of non-communicable disease in low-and middle-income countries Program on the Global Demography of Aging, 2011 http://www.who.int/nmh/publications/best_buys_summary.pdf (cited 07 Mar 2016).

[3] Allen L, Cobiac L, Townsend N Quantifying the global distribution of premature mortality from non-communicable diseases. J Public Health 2017;39:698–703. 10.1093/pubmed/fdx008 28184435

[4] United Nations General Assembly. Addis Abba Action Agenda. A/RES/69/313, 2015.

[5] World Health Organization. Global status report on noncommunicable diseases, 2010 http://www.who.int/nmh/publications/ncd_report2010/en/ (cited 07 Mar 2016).

[6] World Health Organization. First global ministerial conference on healthy lifestyles and noncommunicable disease control. Moscow: The Russian Federation. Prevention and Control of NCDs: Priorities for Investment, 2011 http://www.who.int/nmh/publications/who_bestbuys_to_prevent_ncds.pdf (cited 07 Mar 2016).

[7] Hunter DJ, Reddy KS Noncommunicable diseases. N Engl J Med 2013;369:1336–43. 10.1056/NEJMra1109345 24088093

[8] World Health Organization. Global Health Estimates, 2012 http://www.who.int/healthinfo/global_burden_disease/en/ (cited 07 Mar 2016).

[9] Moher D, Liberati A, Tetzlaff J, et al Preferred reporting items for systematic reviews and meta-analyses: the PRISMA statement. Ann Intern Med 2009;151:264–9. 10.7326/0003-4819-151-4-200908180-00135 19622511

[10] The World Bank. World Bank Country and Lending Groups, 2016 http://data.worldbank.org/about/country-and-lending-groups (cited 07 Mar 2016).

[11] World Health Organization. Global action plan for the prevention and control of noncommunicable diseases 2013-2020. http://www.who.int/nmh/events/ncd_action_plan/en/ (cited 07 Mar 2016).

[12] Fleiss JL The measurement of interrater agreement. Statistical methods for rates and proportions 1981;2:212–36.

[13] Cochrane Effective Practice and Organisation of Care Group. Data Collection Checklist, 2015 http://epoc.cochrane.org/sites/epoc.cochrane.org/files/uploads/datacollectionchecklist.pdf (cited 07 Mar 2016).

[14] Higgins JP, Green S Cochrane handbook for systematic reviews of interventions. 5: Wiley Online Library, 2008.

[15] Higgins JP, Altman DG, Gøtzsche PC, et al The Cochrane Collaboration’s tool for assessing risk of bias in randomised trials. BMJ 2011;343:d5928 10.1136/bmj.d5928 22008217 PMC3196245

[16] Wells GA, Shea B, O’Connell D, et al The Newcastle-Ottawa Scale (NOS) for assessing the quality of nonrandomised studies in meta-analyses, 2000 http://www.ohri.ca/programs/clinical_epidemiology/oxford.asp (cited 10 Jan 2017).

[17] Kaur J, Prasad VM Air nicotine monitoring for second hand smoke exposure in public places in India. Indian J Community Med 2011;36:98 10.4103/0970-0218.84126 21976792 PMC3180954

[18] Nayak NS, Annigeri VB, Revankar DR, et al Secondhand smoke in public places: can Bangalore metropolitan transport corporation be a role model for effective implementation of cigarette and other tobacco products Act, 2003? Indian J Cancer 2010;47:24 10.4103/0019-509X.65316 20622410

[19] Barnoya J, Arvizu M, Jones MR, et al Secondhand smoke exposure in bars and restaurants in Guatemala City: before and after smoking ban evaluation. Cancer Causes Control 2011;22:151–6. 10.1007/s10552-010-9673-8 21046446

[20] Mallikarjun S, Rao A, Rajesh G, et al Role of tobacco warning labels in informing smokers about risks of smoking among bus drivers in Mangalore, India. Asian Pac J Cancer Prev 2014;15:8265–70. 10.7314/APJCP.2014.15.19.8265 25339016

[21] Murukutla N, Turk T, Prasad CV, et al Results of a national mass media campaign in India to warn against the dangers of smokeless tobacco consumption. Tob Control 2012;21:12–17. 10.1136/tc.2010.039438 21508418

[22] Reddy KS, Arora M, Perry CL, Kohli A, et al Tobacco and alcohol use outcomes of a school-based intervention in New Delhi. Am J Health Behav 2002;26:173–81. 10.5993/AJHB.26.3.2 12018753

[23] Naik S, Khanagar S, Kumar A, et al Assessment of effectiveness of smoking cessation intervention among male prisoners in India: A randomized controlled trial. J Int Soc Prev Community Dent 2014;4(Suppl 2):110 10.4103/2231-0762.146213 PMC4278102 25558450

[24] Huque R, Dogar O, Cameron I, et al Children Learning About Second-Hand Smoking: A Feasibility Cluster Randomized Controlled Trial. Nicotine Tob Res 2015;17:1465–72. 10.1093/ntr/ntv015 25634936

[25] Savant SC, Hegde-Shetiya S, Agarwal D, et al Effectiveness of individual and group counseling for cessation of tobacco habit amongst industrial workers in pimpri, pune--an interventional study. Asian Pac J Cancer Prev 2013;14:1133–9. 10.7314/APJCP.2013.14.2.1133 23621201

[26] Jayakrishnan R, Uutela A, Mathew A, et al Smoking cessation intervention in rural kerala, India: findings of a randomised controlled trial. Asian Pac J Cancer Prev 2013;14:6797–802. 10.7314/APJCP.2013.14.11.6797 24377608

[27] Mohlman MK, Boulos DN, El Setouhy M, et al A randomized, controlled community-wide intervention to reduce environmental tobacco smoke exposure. Nicotine Tob Res 2013;15:1372–81. 10.1093/ntr/nts333 23328881 PMC3715387

[28] Anantha N, Nandakumar A, Vishwanath N, et al Efficacy of an anti-tobacco community education program in India. Cancer Causes Control 1995;6:119–29. 10.1007/BF00052772 7749051

[29] Thankappan KR, Mini GK, Daivadanam M, et al Smoking cessation among diabetes patients: results of a pilot randomized controlled trial in Kerala, India. BMC Public Health 2013;13:47 10.1186/1471-2458-13-47 23331722 PMC3560246

[30] Mishra GA, Majmudar PV, Gupta SD, et al Workplace tobacco cessation program in India: A success story. Indian J Occup Environ Med 2009;13:146 10.4103/0019-5278.58919 20442834 PMC2862448

[31] Mishra GA, Kulkarni SV, Majmudar PV, et al Community-based tobacco cessation program among women in Mumbai, India. Indian J Cancer 2014;51:54 10.4103/0019-509X.147474 25526250

[32] Sorensen G, Pednekar MS, Sinha DN, et al Effects of a tobacco control intervention for teachers in India: results of the Bihar school teachers study. Am J Public Health 2013;103:2035–40. 10.2105/AJPH.2013.301303 24028234 PMC3828698

[33] Sorensen G, Gupta PC, Nagler E, et al Promoting life skills and preventing tobacco use among low-income Mumbai youth: effects of Salaam Bombay Foundation intervention. PLoS One 2012;7:e34982 10.1371/journal.pone.0034982 22523567 PMC3327682

[34] Arora M, Tewari A, Tripathy V, et al Community-based model for preventing tobacco use among disadvantaged adolescents in urban slums of India. Health Promot Int 2010;25:143–52. 10.1093/heapro/daq008 20190265

[35] Perry CL, Stigler MH, Arora M, et al Preventing tobacco use among young people in India: Project MYTRI. Am J Public Health 2009;99:899–906. 10.2105/AJPH.2008.145433 19299670 PMC2667859

[36] Nishtar S, Mirza YA, Jehan S, et al Newspaper articles as a tool for cardiovascular prevention programs in a developing country. J Health Commun 2004;9:355–69. 10.1080/10810730490468603 15371087

[37] Subitha L, Soudarssanane MB, Murugesan R Community-based physical activity intervention using principles of social marketing: a demonstration project in Southern India. Natl Med J India 2013;26:12–17. 24066987

[38] Yusuf S, Pais P, Afzal R, et al Effects of a polypill (Polycap) on risk factors in middle-aged individuals without cardiovascular disease (TIPS): a phase II, double-blind, randomised trial. Lancet 2009;373:1341–51. 10.1016/S0140-6736(09)60611-5 19339045

[39] Tian M, Ajay VS, Dunzhu D, et al A Cluster-Randomized, Controlled Trial of a Simplified Multifaceted Management Program for Individuals at High Cardiovascular Risk (SimCard Trial) in Rural Tibet, China, and Haryana, India. Circulation 2015;132:815–24. 10.1161/CIRCULATIONAHA.115.015373 26187183 PMC4558306

[40] Pareek A, Chandurkar NB, Sharma R, et al Efficacy and Tolerability of a Fixed-Dose Combination of Metoprolol Extended Release/Amlodipine in Patients with Mild-to-Moderate Hypertension. Clin Drug Investig 2010;30:123–31. 10.2165/11531770-000000000-00000 20067330

[41] Balasubramanian R, Varadharajan S, Kathale A, et al Assessment of the efficacy and tolerability of a fixed dose combination of atorvastatin 10 mg + metformin SR 500 mg in diabetic dyslipidaemia in adult Indian patients. J Indian Med Assoc 2008;106:464–7. 18975505

[42] Whittle H, Jaffar S, Wansbrough M, et al Observational study of vaccine efficacy 14 years after trial of hepatitis B vaccination in Gambian children. BMJ 2002;325:569 10.1136/bmj.325.7364.569 12228132 PMC124550

[43] Fortuin M, Chotard J, Jack AD, et al Efficacy of hepatitis B vaccine in the Gambian expanded programme on immunisation. Lancet 1993;341:1129–32. 10.1016/0140-6736(93)93137-P 8097813

[44] Chotard J, Inskip HM, Hall AJ, et al The Gambia Hepatitis Intervention Study: follow-up of a cohort of children vaccinated against hepatitis B. J Infect Dis 1992;166:764–8. 10.1093/infdis/166.4.764 1388196

[45] Coursaget P, Leboulleux D, Soumare M, et al Twelve-year follow-up study of hepatitis B immunization of Senegalese infants. J Hepatol 1994;21:250–4. 10.1016/S0168-8278(05)80404-0 7989718

[46] Parham GP, Mwanahamuntu MH, Sahasrabuddhe VV, et al Implementation of cervical cancer prevention services for HIV-infected women in Zambia: measuring program effectiveness. HIV Ther 2010;4:713–22. 10.2217/hiv.10.52 PMC4237284 25419240

[47] Shastri SS, Mittra I, Mishra GA, et al Effect of VIA screening by primary health workers: randomized controlled study in Mumbai, India. J Natl Cancer Inst 2014;106:dju009 10.1093/jnci/dju009 24563518 PMC3982783

[48] Sankaranarayanan R, Nene BM, Shastri SS, et al HPV screening for cervical cancer in rural India. N Engl J Med 2009;360:1385–94. 10.1056/NEJMoa0808516 19339719

[49] Sankaranarayanan R, Esmy PO, Rajkumar R, et al Effect of visual screening on cervical cancer incidence and mortality in Tamil Nadu, India: a cluster-randomised trial. The Lancet 2007;370:398–406. 10.1016/S0140-6736(07)61195-7 17679017

[50] Agarwal SS, Murthy NS, Sharma S, et al Evaluation of a hospital based cytology screening programme for reduction in life time risk of cervical cancer. Neoplasma 1995;42:93–6. 7617084

[51] Vet JN, Kooijman JL, Henderson FC, et al Single-visit approach of cervical cancer screening: see and treat in Indonesia. Br J Cancer 2012;107:772–7. 10.1038/bjc.2012.334 22850550 PMC3425980

[52] Bhatla N, Gulati A, Mathur SR, et al Evaluation of cervical screening in rural North India. Int J Gynaecol Obstet 2009;105:145–9. 10.1016/j.ijgo.2008.12.010 19200539

[53] Sornpaisarn B, Shield K, Cohen J, et al Elasticity of alcohol consumption, alcohol-related harms, and drinking initiation in low- and middle-income countries: A systematic review and meta-analysis. Int J Alcohol Drug Res 2013;2:45–58. 10.7895/ijadr.v2i1.50

[54] Cook WK, Bond J, Greenfield TK Are alcohol policies associated with alcohol consumption in low- and middle-income countries? Addiction 2014;109:1081–90. 10.1111/add.12571 24716508 PMC4107632

[55] De Bruijn A Alcohol marketing practices in Africa: Findings from monitoring exercises in Gambia, Ghana, Nigeria and Uganda. Brazzaville, Congo: World Health Organization African Regional Office, 2011.

[56] World Health Organization. Global status report on alcohol and health. Geneva: World Health Organization, 2014.

[57] The Tobacco Atlas. Our largest objective is to dramatically reduce the consumption of combustible cigarettes, 2016 http://www.tobaccoatlas.org/topic/cigarette-use-globally/ (cited 09 Aug 2016).

[58] Savell E, Gilmore AB, Sims M, et al The environmental profile of a community’s health: a cross-sectional study on tobacco marketing in 16 countries. Bull World Health Organ 2015;93:851–61. 10.2471/BLT.15.155846 26668437 PMC4669733

[59] World Health Organization. WHO report on the global tobacco epidemic, 2015: Raising taxes on tobacco. Geneva: WHO, 2015.

[60] World Health Organization. Non-communicable Disease Progress Monitor 2015. Geneva: WHO, 2015.

[61] Levy DT, Bales S, Lam NT, et al The role of public policies in reducing smoking and deaths caused by smoking in Vietnam: results from the Vietnam tobacco policy simulation model. Soc Sci Med 2006;62:1819–30. 10.1016/j.socscimed.2005.08.043 16182422

[62] Higashi H, Barendregt JJ Cost-effectiveness of tobacco control policies in Vietnam: the case of personal smoking cessation support. Addiction 2012;107:658–70. 10.1111/j.1360-0443.2011.03632.x 21883602

[63] World Health Organization. global progress report on the implementation of the WHO Framework Convention on Tobacco Control. WHO: Geneva, 2010.

[64] Higashi H, Truong KD, Barendregt JJ, et al Cost effectiveness of tobacco control policies in Vietnam. Appl Health Econ Health Policy 2011;9:183–96. 10.2165/11539640-000000000-00000 21506624

[65] Blecher E The impact of tobacco advertising bans on consumption in developing countries. J Health Econ 2008;27:930–42. 10.1016/j.jhealeco.2008.02.010 18440661

[66] Pant NK, Pandey KC, Madabhavi I, et al Evaluation of the Knowledge and Perceptions with Regards to Pictorial Health Warnings on Tobacco Products among Tobacco Users Diagnosed with Head and Neck Carcinoma: a Study from the Kumaon Hills of India. Asian Pacific Journal of Cancer Prevention 2014;15:7891–5. 10.7314/APJCP.2014.15.18.7891 25292083

[67] Eriksen M, Mackay J, Ross H The Tobacco Atlas. 4th edn Atlanta, New York: American Cancer Society, World Lung Foundation, 2012.

[68] Agaku IT, Obadan EM, Odukoya OO, et al Tobacco-free schools as a core component of youth tobacco prevention programs: a secondary analysis of data from 43 countries. Eur J Public Health 2015;25:210–5. 10.1093/eurpub/cku203 25488975

[69] Downs SM, Singh A, Gupta V, et al The need for multisectoral food chain approaches to reduce trans fat consumption in India. BMC Public Health 2015;15:693 10.1186/s12889-015-1988-7 26197873 PMC4511032

[70] Ha DA, Chisholm D Cost-effectiveness analysis of interventions to prevent cardiovascular disease in Vietnam. Health Policy Plan 2011;26:210–22. 10.1093/heapol/czq045 20843878

[71] Mason H, Shoaibi A, Ghandour R, et al A cost effectiveness analysis of salt reduction policies to reduce coronary heart disease in four Eastern Mediterranean countries. PLoS One 2014;9:e84445 10.1371/journal.pone.0084445 24409297 PMC3883693

[72] Asaria P, Chisholm D, Mathers C, et al Chronic disease prevention: health effects and financial costs of strategies to reduce salt intake and control tobacco use. Lancet 2007;370:2044–53. 10.1016/S0140-6736(07)61698-5 18063027

[73] World Health Organization. Reducing salt intake in populations: report of a WHO forum and technical meeting, 5-7 October. Paris, France, 2006 http://www.who.int/dietphysicalactivity/Salt_Report_VC_april07.pdf (cited 10 Jan 2017).

[74] Ohlhorst SD, Slavin M, Bhide JM, et al Use of Iodized Salt in Processed Foods in Select Countries Around the World and the Role of Food Processors. Compr Rev Food Sci Food Saf 2012;11:233–84. 10.1111/j.1541-4337.2011.00182.x

[75] Abioye AI, Hajifathalian K, Danaei G Do mass media campaigns improve physical activity? a systematic review and meta-analysis. Arch Public Health 2013;71:20–4. 10.1186/0778-7367-71-20 23915170 PMC3737034

[76] Lachat C, Otchere S, Roberfroid D, et al Diet and physical activity for the prevention of noncommunicable diseases in low- and middle-income countries: a systematic policy review. PLoS Med 2013;10:e1001465 10.1371/journal.pmed.1001465 23776415 PMC3679005

[77] Lim SS, Gaziano TA, Gakidou E, et al Prevention of cardiovascular disease in high-risk individuals in low-income and middle-income countries: health effects and costs. Lancet 2007;370:2054–62. 10.1016/S0140-6736(07)61699-7 18063025

[78] Ginsberg GM, Lauer JA, Zelle S, et al Cost effectiveness of strategies to combat breast, cervical, and colorectal cancer in sub-Saharan Africa and South East Asia: mathematical modelling study. BMJ 2012;344:e614 10.1136/bmj.e614 22389347 PMC3292522

[79] Allen LN Why is there no funding for Non-Communicable Diseases? J Glo Heal Perspec 2016 http://jglobalhealth.org/article/why-is-there-no-funding-for-non-communicable-diseases/pdf/ (cited 10 Jan 2017).

[80] World Health Organization. A guide to implementation research in the prevention and control of noncommunicable diseases, 2016 http://apps.who.int/iris/bitstream/10665/252626/1/9789241511803-eng.pdf (cited 10 Jan 2017).

