# Supplementary material for: Evaluation of research on interventions aligned to WHO ‘Best Buys’ for NCDs in low-income and lower-middle-income countries: a systematic review from 1990 to 2015
Source: BMJ Glob Health. 2018 Feb 19;3(1):e000535. doi: 10.1136/bmjgh-2017-000535 (PMC5841523; doi:10.1136/bmjgh-2017-000535)
Supplement: Supplementary file 2 [file bmjgh-2017-000535supp002.pdf]

Appendix 1: Search Strategy

Searches conducted on 5th February 2015. Embase, Global Health and Ovid MEDLINE (R) In-Process & Other Non-Indexed Citations and Ovid MEDLINE(R) databases searched via the OVIDSP interface. Web of Science Core Collection database searched via the Thomson Reuters interface.

Search Terms and Strategy

# ▲	Searches
1	Developing Countries/
2	(Africa or Caribbean or West Indies or South America or Latin America or Central America).hw,kf,ti,ab,cp.
3	(Benin or Burkina Faso or Burkina Fasso or Upper Volta or Burundi or Urundi or Central African Republic or Chad or Comoros or Comoro Islands or Comores or Mayotte or Congo or Democratic Republic of Congo or Republic of Zaire or Zaire or Eritrea or Ethiopia or Gambia or The Gambia or Guinea or Guinea Bissau or Liberia or Madagascar or Malagasy Republic or Malawi or Nyasaland or Mali or Mozambique or Niger or Rwanda or Ruanda or Sierra Leone or South Sudan or Tanzania or Togo or Togolese or Togolese Republic or Uganda or Zimbabwe or Cape Verde or Cabo Verde or Cameroon or Cameron or Camerons or Cote d'Ivoire or Ivory Coast or Ghana or Guiana or Guyana or Gold coast or Kenya or Lesotho or Basutoland or Mauritania or Nigeria or Sao Tome or Principe or Senegal or Swaziland or Zambia or Zimbabwe or Rhodesia or Cambodia or Khmer Republic or Kampuchea or Republic of Korea or North Korea or Korea or Kiribati or Laos or Lao or Lao Democratic Peoples Republic or Lao PDR or Federated States of Micronesia or Micronesia or Papua New Guinea or Philippines or Phillipines or Philipines or Samoa or Samoan Islands or Solomon Islands or Vanuatu or New Hebrides or Vietnam or Viet nam or Afghanistan or Somalia or Djibouti or French Somaliland or Egypt or United Arab Republic or Morocco or Ifni or Sudan or Syria or Syrian Arab Republic or West Bank and Gaza or Palestine or Yemen or Republic of Yemen or Bolivia or Guatemala or Guyana or Guiana or Honduras or Nicaragua or Haiti or El Salvador or Nepal or Bangladesh or Bhutan or India or Indonesia or Myanmar or Burma or Myanma or Pakistan or Sri Lanka or Timor leste or East Timor or East Timur or Armenia or Armenian or Georgia or Georgian Republic or Georgia Republic or Kosovo or Kyrgyz Republic or Kyrgyzstan or Kirghizia or Kyrgyz Republic or Kirghiz or Kirgizstan or Moldova or Moldovia or Tajikistan or Tadzhikistan or Tadjikistan or Tadzhik or Ukraine or Uzbekistan or Uzbek or Middle East).hw,kf,ti,ab,cp.
4	((developing or less* developed or under developed or underdeveloped or middle income or low* income or underserved or under served or deprived or poor*) adj (countr* or nation? or state? or population? or world)).ti,ab.
5	((developing or less* developed or under developed or underdeveloped or middle income or low* income) adj (economy or economies)).ti,ab.
6	(low* adj (gdp or gnp or gross domestic or gross national)).ti,ab.
7	(low adj3 middle adj3 countr*).ti,ab.
8	(lmic or lmics or third world or lami countr*).ti,ab.
9	transitional countr*.ti,ab.
10	1 or 2 or 3 or 4 or 5 or 6 or 7 or 8 or 9
11	cardiovascular diseases/ or heart diseases/ or vascular diseases/ or cerebrovascular diseases/
12	exp Myocardial Ischemia/
13	Heart Failure/
14	exp brain ischemia/ or exp stroke/
15	exp Diabetes Mellitus, Type 2/

16 lung diseases, obstructive/ or exp pulmonary disease, chronic obstructive/

17 exp *Neoplasms/

18 ((cardiovascular or cardio-vascular) adj3 disease*).ti,ab.

19 ((cardiovascular or cardio-vascular) adj3 (event* or outcome* or risk*)).ti,ab.

20 ((coronary or heart or myocard*) adj3 disease*).ti,ab.

21 ((coronary or heart or myocard*) adj3 (event* or outcome* or risk*)).ti,ab.

22 ((ischaemic or ischemic or ischaemia or ischemia) adj3 disease*).ti,ab.

23 ((ischaemic or ischemic or ischaemia or ischemia) adj3 (event* or outcome* or risk*)).ti,ab.

24 myocardial infarct*.ti,ab.

25 ((cerebrovascular or vascular) adj3 disease*).ti,ab.

26 ((cerebrovascular or vascular) adj3 (event* or outcome* or risk*)).ti,ab.

27 stroke.ti,ab.

28 heart failure.ti,ab.

29 diabet*.ti.

30 ((type 2 or type ii or noninsulin dependent or non insulin dependent or adult onset or maturity onset or obes*) adj2 diabet*).ti,ab.

31 (niddm or t2dm or tiidm).ti,ab.

32 (chronic adj2 (lung or pulmonary)).ti,ab.

33 chronic obstructive pulmonary disease.ti,ab.

34 (neoplas* or cancer* or carcinoma* or tumor* or tumour* or malignan* or leukaemia or leukemia or lymphoma?).ti,ab.

35 11 or 12 or 13 or 14 or 15 or 16 or 17 or 18 or 19 or 20 or 21 or 22 or 23 or 24 or 25 or 26 or 27 or 28 or 29 or 30 or 31 or 32 or 33 or 34

36 Taxes/ and ("Tobacco Use"/ or exp "Tobacco Use Cessation"/ or drinking behavior/ or exp alcohol drinking/ or exp dietary fats/ or Sodium Chloride/)

37 (tax or taxes or taxing or taxation).ti.

38 ((food? or diet* or vegetable? or fruit? or sugar* or fat or fats or sucrose or candy or sweet* or snack* or fastfood? or junkfood?) and (tax or taxes or taxing or taxation)).ti,ab.

39 ((smok* or tobacco) and (tax or taxes or taxing or taxation)).ti,ab.

40 ((alcohol or drinking) and (tax or taxes or taxing or taxation)).ti,ab.

41 ((smok* or tobacco) and (subsidy or subsidies or incentiv* or voucher?)).ti,ab.

42 ((alcohol or drinking) and (subsidy or subsidies or incentiv* or voucher?)).ti,ab.

43 (Social Control, Formal/ or Legislation/) and ("Tobacco Use"/ or exp "Tobacco Use Cessation"/ or Tobacco Industry/ or Tobacco Smoke Pollution/ or drinking behavior/ or exp alcohol drinking/ or exp dietary fats/ or fast foods/ or Sodium Chloride/)

44 smoke-free policy/

45 exp Nutrition Policy/

46 ((smoke or smoking or tobacco) adj2 (ban or bans or banned or free)).ti,ab.

47 ((smoke or smoking or tobacco) and (legislat* or law? or regulation or regulatory)).ti,ab.

48 ((alcohol or drinking) and (legislat* or law? or regulation or regulatory)).ti,ab.

49 exp Trans Fatty Acids/

50 Social Marketing/

51 (Marketing/ or advertising as topic/ or Mass Media/ or product packaging/ or product labeling/) and ("Tobacco Use"/ or exp "Tobacco Use Cessation"/ or drinking behavior/ or exp alcohol drinking/ or exp Diet/ or food/ or exp dietary fats/ or fast foods/ or fruit/ or vegetables/ or Sodium Chloride, Dietary/ or Beverages/ or exp Exercise/ or Motor Activity/ or health behavior/ or risk reduction behavior/)

52 food packaging/ or food labeling/

53	((food? or diet* or vegetable? or fruit? or sugar* or fat or fats or sucrose or candy or sweet* or snack* or fastfood? or junkfood?) and (marketing or adverti?ing or sponsorship? or label* or pack*)).ti,ab.
54	((smok* or tobacco) and (marketing or adverti?ing or sponsorship? or label* or pack*)).ti,ab.
55	exp alcohol drinking/ and retail.mp
56	exp alcohol drinking/ and restriction.mp
57	((alcohol or drinking) and (marketing or adverti?ing or sponsorship? or label* or pack*)).ti,ab.
58	(salt adj3 (intake or reduc* or lower*)).ti,ab.
59	Health Promotion/ and (Neoplasms/ or Obesity/ or Diabetes Mellitus, Type 2/ or Smoking/ or Risk Factors/ or Cardiovascular Diseases/ or Hypertension/)
60	Drug therapy, combination and (Diabetes Mellitus, Type 2/ or Cardiovascular Diseases/ or Hypertension/)
61	Patient Education as Topic/ or Counseling/ or Patient Compliance/ or Motivational Interviewing/ and (Neoplasms/ or Obesity/ or Diabetes Mellitus, Type 2/ or Smoking/ or Risk Factors/ or Cardiovascular Diseases/ or Hypertension/)
62	Health Education/ or Health Knowledge, Attitudes, Practice/ and (Life Style/ or Food Habits/ or Diet/ or Health Behavior/ or Obesity/ or Motor Activity/
63	Hydroxymethylglutaryl-CoA Reductase Inhibitors/ or Simvastatin/ or Aspirin/ or Metformin/ or Adrenergic beta-Antagonists/ or Propranolol/
64	Food legislation/ and (dietary fats/ or sodium/)
65	Food supply/ and (dietary fats/ or sodium/)
66	Papanicolaou Test/
67	Mass Screening/ and (Uterine Cervical Neoplasms/ or Cervical Intraepithelial Neoplasia/)
68	((cervical or pap) adj3 screen*).ti,ab.
69	Hepatitis B Vaccines/
70	((hepatitis b or hep b) adj3 (vaccin* or immuni?ation or immuni?e)).ti,ab.
71	*Aspirin/
72	Primary Prevention/ and Aspirin/
73	(aspirin or acetylsalicylic acid).ti,ab.
74	best Buys.mp
75	or/36-74
76	10 and 35 and 75
77	(intervention* or pilot or feasability).ti.
78	evaluation studies as topic/ or program evaluation/
79	(program* or model*).ti.
80	(program* adj2 (evaluat* or monitor* or implement* or develop*)).ti,ab.
81	((econ* or cost* or socioecon*) adj3 model*).ti,ab.
82	77 or 78 or 79 or 80 or 81
83	76 and 82

Appendix 2: Extracted data variables

- Authors
- Reference
- Year
- Country
- Sample description
- Study design
- Age mean
- Age range
- Sex
- Sample frame size
- N included
- N responders/n with complete follow up information
- Source of funding
- Exposures and definition
- Subgroups
- Outcomes and definition
- Crude and adjusted results
- P values
- 95%CIs
- Adjusted variables

Appendix 3: Risk of Bias scoring

	Selection				Comparability	Outcome/Exposure		
**Case-Control[1]**	Is Case Definition Adequate?	Representativeness of the Cases	Selection of Controls	Definition of Controls	Comparability of cases and controls on basis of design/analysis	Ascertainment of Exposure	Non-Response Rate	
**Cohort[1]**	Representativeness of the exposed cohort	Selection of the non-exposed cohort	Ascertainment of exposure	Demonstration that outcome of interest was not present at start of study	Comparability of cohorts on the basis of the design or analysis	Assessment of outcome	Was follow-up long enough for outcomes to occur	Adequacy of follow-up of cohorts
**Cross-sectional [2]**	Representativeness of the sample	Sample Size	Non-respondents	Ascertainment of the exposure	Comparability of subjects in different outcome groups (control for confounding)	Assessment of the outcome	Statistical test is appropriate	
**Interrupted time series [3]**	shape of intervention prespecified (they say what they expect to happen)	Intervention independent of other changes/confounders/historic changes	Intervention did not affect data collection/data collection method same pre and post	Allocation concealment (blind or objective outcome assessment)	Incomplete data adequately addressed	all outcomes mentioned in methods are reported in results	free from other sources of bias e.g. seasonality	
**RCT [4]**	Random sequence generation (selection bias)	Allocation concealment (selection bias)	Blinding of participants and personell (performance bias)	Blinding of outcome assessment (detection bias)	Incomplete oucome data (reporting bias)	Selective reporting (reporting bias)	Other bias	
Score	**1**	**1**	**1**	**1**	**1**	**1**	**1**	**1**

Low = 0-2 **Medium = 3-5** **High = 6-8**

1: Based on Newcastle Ottawa Scale
2: Based on Newcastle Ottawa Scale
3: Based on Cochrane Collaboration’s tool for assessing risk of bias in non-randomised trials
3: Based on Cochrane Collaboration’s tool for assessing risk of bias in randomised trials

Appendix 4: Grading level of evidence

This scheme is taken from:

http://jama.jamanetwork.com/public/instructionsForAuthors.aspx#GeneralInformation

and based on:

OCEBM Levels of Evidence Working Group.* "The Oxford Levels of Evidence 2"

Oxford Centre for Evidence-Based Medicine. http://www.cebm.net/index.aspx?o=5653

* OCEBM Levels of Evidence Working Group: Jeremy Howick, Iain Chalmers (James Lind Library), Paul Glasziou, Trish Greenhalgh, Carl Heneghan, Alessandro Liberati, Ivan Moschetti, Bob Phillips, Hazel Thornton, Olive Goddard and Mary Hodgkinson

Quality Rating Scheme for Studies and Other Evidence

1	Properly powered and conducted randomized clinical trial; systematic review with meta-analysis
2	Well-designed controlled trial without randomization; prospective comparative cohort trial
3	Case-control studies; retrospective cohort study
4	Case series with or without intervention; cross-sectional study
5	Opinion of respected authorities; case reports
