# Supplementary material for: Evaluation of research on interventions aligned to WHO ‘Best Buys’ for NCDs in low-income and lower-middle-income countries: a systematic review from 1990 to 2015
Source: BMJ Glob Health. 2018 Feb 19;3(1):e000535. doi: 10.1136/bmjgh-2017-000535 (PMC5841523; doi:10.1136/bmjgh-2017-000535)
Supplement: Supplementary file 3 [file bmjgh-2017-000535supp003.pdf]

Study	Setting	Design	Funding	Quality scores (See Appenidix 3)								Quality
				Selection				Comparability	Outcome/Exposure			
**Tobacco Bans in public places**												
Kaur 2011	India	Longitudinal passive air monitoring	John Hopkins Bloomberg School of Public Health	1	1	1	0	0	0	1		Medium
Nayak 2010	India	Retrospective cross-sectional survey	American cancer Society	1	1	0	1	1	1	1		High
Barnoya 2011	Guatemala	Longitudinal passive nicotine monitoring and surveys	Cardiovascular Unit of Guatemala (UNICAR); Unit of Guatemala (UNICAR); American ancer Society; Flight Attendant medical research Institite	1	1	1	1	1	1	1		High
**Tobacco warnings and information**												
Mallikarjun 2014	India	Retrospective cross-sectional survey	Not reported	1	1	1	1	0	1	1		High
**Tobacco mass media**												
Murukutla 2012	India	retrospective cross-sectional survey	Bloomberg Philanthropies and World Lung Foundation	1	0	1	0	1	1	1		Medium
**Tobacco group programmes**												
Reddy 2002	India	Three arm RCT with before/after cross sectional surveys	Fogarty International research Collaboration Award, NIH	1	1	0	0	0	1	1		Medium
Naik 2014	India	Two arm RCT	No formal funding	1	0	0	1	1	1	1		High
Huque 2015	Bangladesh	Three arm cluster RCT	Not reported	1	1	0	1	1	1	1		High
Savant 2013	India	Three arm RCT	Not reported	1	0	0	0	1	0	1		Medium
Jayakrishnan 2013	India	Two-arm RCT	Not reported	1	1	0	0	1	1	1		Medium
Mohlman 2013	Egypt	Cluster RCT	The Fogarty International Center of the U.S. National Institutes of Health	1	0	0	1	1	0			High
Anantha 1995	India	Cluster RCT	Indian Council of Medical Research	1	1	1	1	1	0	1		High
Thankappan 2013	India	Two-arm RCT	Fogarty International Centre of the US National Institutes of Health	1	1	0	1	1	0	1		Medium
Mishra 2014	India	Longitudinal	Not reported	1	0	1	1	0	0	1		Medium
Sorensen 2012	India	Cluster RCT	Not reported	1	1	1	1	1	0	1		High
Sorenson 2013	India	Cluster RCT	National Cancer Institute	1	1	0	1	1	0	1		High
Mishra 2009	India	Longitudinal	Not reported	1	1	1	1	0	1	1	1	High
Arora 2010	India	Cluster RCT	Initiative for Cardiovascular Health Research in Developing Countries	1	0	0	0	0	1	1		Medium
Perry 2009	India	Cluster step RCT	Fogarty International Centre of the US National Institutes of Health	1	1	1	1	1	0	1		High
**Individual smoking cessation programmes**												
Savant 2013	India	Three arm RCT	Not reported	1	0	0	0	1	0	1		Medium
**Physical activity mass media**												
Nishtar 2004 - Physical activity component	Pakistan	Retrospective cross-sectional survey	DFID, CIDA	1	1	1	1	1	1	1		High
Subitha 2013	India	Cohort study	Not reported	1	1	1	1	1	1	1		High
**Diet mass media**												
Nishtar 2004 - Diet component	Pakistan	Retrospective cross-sectional survey	DFID, CIDA	1	1	1	1	1	1	1		High

**Counselling and poly-drug therapy for high-risk groups (CVD prevention)**												
Yusuf 2009	India	Phase II RCT drug trial	Cadila Pharmaceuticals, Ahmedabad, India	1	1	1	1	1	1	1		High
Tian 2015	India	Cluster RCT	US National Heart, Lung, and Blood Institute, National Institutes of Health, Department of Health and Human Services	1	1	1	1	1	0	1		High
Pareek 2010	India	Phase II RCT drug trial	Ipca Laboratories Limited, Mumbai, India	1	1	1	1	1	1	1		High
**Poly-antidiabetic therapy and treatment of hypertention in diabteics**												
Balasubramanian 2008	India	Phase II drug trial	Not reported	1	0	1	1	1	1	0		Medium
**Hepatitis B immunization to prevent liver cancer**												
Whittle 2002	Gambia	Cohort	Not reported	1	0	1	1	1	1	1	1	High
Fortuin 1993	Gambia	Cohort	Direzione Generale per la Cooperazione allo Sviluppo of the Ministry of Foreign Affairs of Italy and by the Medical Research Council of Sweden. Vaccine was donated by Merck, Sharp and Dohme.	1	1	1	1	1	1	1	1	High
Chotard 1992	Gambia	Cohort	Grant from the Department for Cooperation and Development of the Ministry of Foreign Affairs of Italy. The vaccine for the study was donated by Merck, Sharp and Dohme.	1	1	1	1	0	1	0	0	Medium
Coursaget 1994	Senegal	Cohort	Ministre de la Cooperation (France), The Secretariat d'Etat la Recherche Scientifique et Technique (Senegal), National de la Sante et de la Recherche M6dicale(France), The Commission of European Communities (Belgium), The Institute of Virology of Tours (France), and the Fondation de France (France).	1	1	1	1	1	1	1	1	High
**Cervical cancer screening and treatment**												
Parham 2010	Zambia	Longitudinal programme evaluation using data modelling	US Centers for Disease Control and Prevention through the President's Emergency Plan for AIDS Relief (PEPFAR) and the Fogarty International Center and National Cancer Institute of the US National Institutes of Health.	0	0	1	0	0	1	1		Medium
Shastri 2014	India	Two-arm cluster RCT	National Institutes of Health (National Cancer Institute) grant award (5R01CA074801) and intramural grant from Tata Memorial Centre. Treatment costs were supported by the Women's Cancer Initiative, India.	1	1	0	1	1	1	1		High
Sankaranarayanan 2009	India	Cluster four-arm RCT	Bill and Melinda Gates Foundation through the Alliance for Cervical Cancer Prevention									
Sankaranarayanan 2007	India	Cluster two-arm RCT	Bill & Melinda Gates Foundation through the Alliance for Cervical Cancer Prevention (ACCP), Seattle, USA.	1	1	0	1	0	1	1		Medium
Agarwal 1995	India	Cohort program evaluation	Not reported	1	1	1	0	0	1	1		Medium
Vet 2012	Indonesia	Cohort programme evaluation	The Female Cancer Foundation, Leiden University Medical Centre, the Netherlands	1	1	1	1	0	1	0	0	Medium
Bhatla 2009	India	Longitudinal	Bill and Melinda Gates Foundation	1	1	0	1	0	1	0		Medium
